# Prevalence of Anterior Dental Trauma and Its Associated Factors among Preschool Children Aged 3–5 Years in Khartoum City, Sudan

**DOI:** 10.1155/2018/2135381

**Published:** 2018-05-24

**Authors:** Alaa Gamaleldin Sulieman, Elhadi Mohieldin Awooda

**Affiliations:** Department of Endodontics, Faculty of Dentistry, University of Medical Sciences and Technology, Khartoum, Sudan

## Abstract

**Background:**

Traumatic dental injury (TDI) is a challenging public health problem. Its incidence and prevalence vary within countries, states, and different social groups.

**Aim:**

The purpose of this study was to determine the prevalence of traumatic dental injuries in primary incisors among 3–5-year-old Sudanese preschool children and associated factors such as age, sex, and size of overjet and anterior open bite.

**Materials and Methods:**

Descriptive cross-sectional study was carried out among 600 preschool children (3–5 years old) selected by multistage cluster technique from preschools located in Khartoum city, Sudan. The data regarding age, sex, causes, and treatment of TDI were collected from the mothers through structured interview questionnaire. Children were examined in an upright position, using mouth mirror and torch. A single examiner assessed the type of trauma, the tooth involved, and overbite/overjet. The data were analyzed statistically through descriptive analysis, and the chi-square test was used to compare between different variables with *P* < 0.05.

**Results:**

The prevalence of traumatic dental injuries (TDIs) was 18.5%. Enamel fractures were the most common type (74.8%), followed by enamel and dentin (11.7%). The maxillary central incisors were the most commonly affected teeth, and the home was most common place for TDI. The prevalence rate was 45% in boys and 55% in girls with a statistically significant difference (*P*=0.02).

**Conclusions:**

The prevalence of traumatic dental injuries to the primary anterior teeth among the Sudanese preschool children was relatively high (18.5%). Factors such as overjet size, overbite size, and lip competency were not significantly correlated with dental trauma among the studied population.

## 1. Introduction

Traumatic dental injury (TDI) is a challenging public health problem to professionals, and it has been seriously neglected [1]. The incidence of dental trauma among the primary school children is an emerging dental health problem. Epidemiological data showed a wide variation in the prevalence of dental injuries in preschool children [2–4]. Several studies revealed that the prevalence of traumatic injuries in children has increased during the last few decades [5, 6]. Dental trauma can result in displacement, fracture, or loss of tooth [4]. The consequence of traumatic injuries to primary teeth includes alteration in physical appearance, speech defects, and emotional impacts, thus affecting the child's quality of life [7, 8]. TDI to primary teeth may eventually create problems to the underlying permanent teeth, such as hypoplasia, discoloration, delay eruption time, and tooth malformation [9, 10]. Preschool children are more prone to TDI due to their poor stability, passive reflexes, and indefinite movements [3, 10]. Predisposing factors to TDI include physical features such as increased incisal overjet, open bite, protrusion, and lip incompetence [8].

In many countries, high cost, low standard of living, and lack of knowledge regarding urgent treatment of dental trauma may result in delaying the treatment [8, 11]. Studies of TDIs among the preschool children are of paramount importance, as individuals with previous trauma in the primary dentition are prone to further trauma in the permanent dentition [12].

A recent meta-analysis study on February 2018 through literature search (publication years 1996–2016 of the prevalence of TDIs) aimed at covering as many countries, communities, and ethnic groups as possible. They showed that more than one billion living people have had TDI [13]. Research studies on TDIs among the Sudanese population are not well documented to be part of the global frequency, so data from the Sudan are needed to achieve high generalizability. The objectives of this study were to determine the prevalence of TDI among the Sudanese preschool children aged 3–5 years and to assess the relation among associated factors such as overbite, overjet, age, gender, and site to the severity of TDI.

## 2. Materials and Methods

A cross-sectional study was carried out on children aged 3–5 years, of both sexes, living in Khartoum city. The inclusion criteria were the child being healthy and of Sudanese nationality attending a public or private preschools. The exclusion criteria included children who experienced significant dental anxiety and those who were identified by the preschool authority to have medical or intellectual problems. Data collection began on November 2016 over a period of two months. The sample size was determined after obtaining the total number of preschools in Khartoum locality, the number of public preschools was 13, and the number of private preschools was 507 with a total number of children within these schools compromising 23,000. We estimated that a minimum sample size of 585 children was required to achieve the level of precision with a standard error of 4% or less and the 95% confidence interval level and a prevalence of dental trauma of 50% used for the calculation. The decision to use a prevalence of 50% was due to the lack of information of the actual published prevalence of TDI among preschool children in Sudan. However, any calculation using a different figure than 50% would require a smaller sample size to achieve the same precision [14]. The number was rounded to 600.

Ethical clearance was obtained from the Ethical Committee of the University of Medical Science and Technology. Local authorities (Ministry of Health and Ministry of Education in Khartoum state) provided the necessary information for the construction of a sample frame including names, locations of preschools in Khartoum, their addresses, contact information, and the total number of children in the locality. Permission to examine the children was obtained from the school authorities concerned. A letter was handed to all parents explaining the objectives of the study and requesting the acceptance of voluntary participation themselves to answer the questionnaire and their children to be examined for the presence of TDI. Those who agreed to participate were signed informed written consent.

Data were collected through intraoral examination of eligible children and their mother's response to a self-administered closed-ended questionnaire including demographic data, etiology, localization, place, time elapsed between injury and treatment, and the treatment provided. The data were collected in the classrooms, and a single examiner, who is the first author, performed the clinical examination. The examined child was seated in classroom chair in the upright position; mouth mirror and torch were used to enhance visualization. One of the parents or schoolteachers sometimes helped to carry very young children. A sample of 15 children was used to train and to calibrate the examiner and test the feasibility of the intraoral visual examination, diagnosis of TDI, and the interview procedures. Training was performed with the help of an experienced endodontist and paedodontist from the University of Medical Sciences and Technology. No changes were made on the methods previously proposed, and the first author carried out all the procedure.

The criteria for the diagnosis of anterior dental trauma were assessed according to the method used by Andreasen et al. [15]. The number of injured teeth, type of trauma, type of tooth, and location in the upper or lower jaw were recorded. The presence of anterior open bite was assessed based on the criterion of lack of vertical overlap of the incisors in the occlusal position [9]. The overjet size was measured in millimeters by a metal ruler. Root fracture was not recorded, and no intraoral radiographs were taken. The data were subjected to simple descriptive analysis, and the statistical analysis was performed by SPSS version 21 (SPSS Inc., Chicago, USA). Statistical significance for the association between the occurrence of dental trauma and the distribution of dental injuries by age, gender, children's incisal overjet size, and overbite size was determined using the chi-square test with the level of statistical significance set at *P* < 0.05.

## 3. Results

A total of 600 children aged 3–5 years and all attending preschools in Khartoum locality, Sudan, were included in this study; the overall children affected by TDI were 111 with the prevalence of 18.5%. The prevalence rate was 45% in boys and 55% in girls, and a statistical significant difference was found between them (*P*=0.02).

The maxillary central incisors were the most common teeth affected by trauma (54.9%), the right maxillary central incisor accounted for 35.1% of the injuries, and the left maxillary central incisor accounted for 19.8% followed by the maxillary lateral incisors (32.4%); meanwhile, the percentage of mandibular incisors was 6.3%, and the least affected tooth was found to be the canine (Table 1).

Enamel fracture accounted for 74.8% and was the most common type of TDI in maxillary and mandibular teeth, followed by fractures involving both enamel and dentin, while complete avulsion affected only 9.9% as displayed in Table 2. TDIs occurred at home accounted for 71.2%. Tripping and falling were the most common causes of TDI with a percentage of 63.1% (Table 3). Regardless of the dental injuries, we found that 95.5% of the subjects did not receive any dental treatment or control of the problem. There is no statistical significant difference between the size of overjet and overbite with occurrence of TDI as seen in Tables 4 and 5.

## 4. Discussion

The most common type of facial injuries is traumatic dental injury accounting for as high as 18% of the all facial injuries [16]. This cross-sectional survey identified the prevalence of dental trauma to the primary anterior teeth in 3–5-year-old preschool children in Khartoum city. In this city, there are no clear variations in socioeconomic status; the city is a melting pool of different ethnic groups coming from other states and regions in the Sudan, so with some cautions, we can generalize the results to the whole Sudan. The prevalence of TDI was higher when compared to another study [17] and lower when compared to most studies of trauma to primary teeth [18]. However, it is difficult to compare the prevalence figures found in different studies due to lack of uniformity in selected population, dental examination procedure, diagnostic criteria, and age groups [19].

Contradicting the majority of previous studies [20–22], which reported a higher frequency of dental trauma among boys than among girls, our study results are consistent with the results of the study done by Rai et al. [23] and Vuletić et al. [5] that girls tend to experience more dental injuries than boys, while the results of other studies revealed no statistically significant differences [3, 17]. However, some studies showed that there is an increase in TDI among girls and this increasing trend of dental trauma among girls is probably because of their increasing participation in sports or physical activities formerly practiced by boys only [24].

Majority of them have enamel fracture; this finding corresponds with previous research studies carried out on different populations [3, 18, 25, 26]. Other studies have reported different results; one such study, which surveyed Turkish children, found that avulsion and crown fractures were the most common injuries [27]. Others reported subluxation and avulsions as the most frequent types of dental trauma [28, 29]. The difference in the findings of these different studies may be due to the population being examined.

Similar to the results of other studies [4, 18], traumatic dental injuries occur more in the maxilla than in the mandible. This may be due to relative prominence of the maxillary anterior teeth. The maxillary central incisors are the teeth most commonly affected, similar to what was obtained by previous studies [3, 4]. The reason may be due to its central position which is more prone to direct assault. In addition, the upper central incisors are generally more proclined than the lower centrals and tend to be the first to receive a direct blow [3]. Also, the upper jaw is fixed to the skull which makes it rigid, while the lower jaw, being a flexible part, tends to reduce the impact forces directed on the lower anterior teeth by movement [30].

The majority of TDIs occurred at home and at school during a physical activity that lead to fall, as demonstrated in this study; 63.1% of dental trauma occurred due to a fall followed by a collision with an object or person. Our results were consistent with the literature that reported the most common cause of injury was a fall and the home being the commonest place [18, 31]; however, some studies concluded that TDIs occur most common in a field/playground or outdoors [27, 32].

Different studies have found an association between an overjet >3 mm and inadequate lip coverage and the occurrence of dental trauma in permanent teeth [3, 8, 11, 18, 31]. A few studies have shown a relationship in deciduous teeth [26, 33, 34]. Our results reveal that there is no significant relation between increased overjet and overbite with TDI among preschoolers. The reason behind this inconsistency cannot be justified, and further studies are needed to prove otherwise.

Relatively high prevalence of TDIs in our study could be due to the increased number of private schools when compared to government schools. This increases the risk of subjecting children to more trauma. As most of the private schools do not follow the standard of an ideal school environment and they build or rent small area in floor building, the space is limited for the children to play free, which leads to crashes with each other. However, this assumption contradicted the result made by Singh et al., where the prevalence of TDIs among preschool children was low (4.10%) and they interpreted due to the relative lack of outdoor activities and more emphasis on education [19].

Majority of traumatized subjects (95.5%) did not seek or receive any dental treatment, and similar result obtained by others [35, 36]. It revealed a poor perception of parents and caregivers toward child's oral health and a lack of awareness regarding TDI to primary teeth. A further study is needed to verify failure of seeking dental trauma treatment to primary teeth.

The prevention of trauma and the need for treatment following TDIs are very important to avoid possible unfavorable consequences that may precipitate [10, 37]. More interestingly and importantly, the report of the study by Awad et al. revealed a low level of knowledge among primary schoolteachers regarding the management of TDIs [38]. As our results showed, most of the trauma happened at home and school; public health care services, as well as primary and preschools, could be the target of educational campaigns directed at parents/teachers to increase their knowledge and practice in emergency management of TDIs.

## 5. Conclusion

The prevalence of traumatic dental injuries to the primary anterior teeth among Sudanese preschool children was relatively high (18.5%). Girls being more affected than boys, enamel fracture was the most common type, and maxillary central incisors were the most affected; no association was found between TDI, overbite size, and overjet size.

## Figures and Tables

**Table 1:** Distribution of the study sample according to the type of the traumatized tooth.

	*N*	%
Right maxillary central incisor	39	35.1
Right maxillary lateral incisor	24	21.6
Right maxillary canine	3	2.7
Left maxillary central incisor	22	19.8
Left maxillary lateral incisor	12	10.8
Left maxillary canine	1	0.9
Left mandibular central incisor	4	3.6
Left mandibular lateral incisor	1	0.9
Left mandibular canine	3	2.7
Right mandibular central incisor	2	1.8
Total	111	100.0

**Table 2:** Distribution of the study sample according to the type of dental trauma to the anterior teeth.

	Code	*N*	%
1	No injury seen	1	0.9
2	Treated dental injury	1	0.95
3	Enamel fracture only	83	74.8
4	Enamel/dentin fracture	13	11.7
5	Enamel/dentin and pulp injury	2	1.8
6	Missing tooth due to trauma	11	9.9
7	Excluded tooth	0	0
	Total	111	100

**Table 3:** Distribution of the study sample according to the cause of anterior dental trauma.

Type	*N*	%
Trips and falls	70	63.1
Collision with objects or person	15	13.55
Bicycles	0	0
Road traffic accident	0	0
Violence or fights	2	1.8
Do not remember	24	21.6
Other (specify)	0	0
Total	111	100.0

**Table 4:** The relationship between traumatic dental injury and overbite size among the studied preschool children.

Overbite		TDI		Total	*P* value
		Yes	No		
Normal	Count	107	459	566	0.681^*∗*^ Pearson's *R* (0.017)
	% of total	17.8%	76.5%	94.3%	
Open bite	Count	4	30	34	
	% of total	0.7%	5.0%	5.7%	
Cross bite	Count	0	0	0	
	% of total	0	0	0	
Total	Count	111	489	600	
	% of total	18.5%	81.5%	100.0%	

TDI: traumatic dental injury, ^*∗*^*P* > 0.05 indicates no significant relation between TDI and overbite.

**Table 5:** The relation between traumatic dental injury and overjet size among the studied preschool children.

Size of overjet		TDI		Total	*P* value
		Yes	No		
0–3 mm	Count	109	477	586	0.298^*∗*^ Pearson's *R* (0.043)
	% of total	18.2%	79.5%	97.7%	
3–6 mm	Count	2	12	14	
	% of total	0.3%	2.0%	2.3%	
More than 6 mm	Count	0	0	0	
	% of total	0%	0%	0%	
Total	Count	111	489	600	
	% of total	18.5%	81.5%	100.0%	

^*∗*^ *P* value ≥ 0.05 indicates no significant difference between TDI and overjet size.

## Data Availability

The data used to support the findings of this study are available from the corresponding author upon request.
